# Bivalent virus-like particles expressing SPECT1 and CSP trigger pre-erythrocytic malaria immunity and protect against transgenic *Plasmodium falciparum* sporozoite challenge in mice

**DOI:** 10.3389/fimmu.2026.1790309

**Published:** 2026-05-14

**Authors:** Gulbuse Turan, Ekta Mukhopadhyay, Adam Truby, Kseniia Fedorova, Marco Polo Peralta Alvarez, Naif Khalaf Alharbi, Adrian V. S. Hill, Ahmed M. Salman

**Affiliations:** The Jenner Institute, Nuffield Department of Medicine, University of Oxford, Oxford, United Kingdom

**Keywords:** Matrix-M, LMQ, malaria, NANP, parasite, SMNP, SPECT-1, VLP

## Abstract

**Introduction:**

The decline in protective antibody titers and efficacy over time of the circumsporozoite protein (CSP)-based RTS,S/AS01 and R21/Matrix-M vaccines highlights the need for improved vaccines. Sporozoite microneme protein essential for cell traversal-1 (SPECT-1) is a conserved *Plasmodium falciparum* (*Pf*) antigen that plays a key role in parasite movements while traversing host cells. Targeting SPECT-1 as an addition to the CSP in the same vaccine may enhance immune response and protection.

**Methods:**

We engineered a series of recombinant Hepatitis B surface antigen (HBsAg) virus-like particles (VLPs) displaying *Pf*SPECT-1 alone or in combination with NANP repeats and C-terminal region of *Pf*CSP. These monovalent and bivalent VLP vaccine candidates were evaluated alongside R21 in a prime-boost regimen which was followed by challenge with transgenic *P. berghei* parasite expressing the same *P. falciparum* antigens. We also assessed the quality of the antibody response following vaccination. Based on the antibody titers and protective efficacy, one bivalent VLP construct was selected for further evaluation with different adjuvant formulations.

**Results:**

All bivalent VLPs formed to display antigenic epitopes that were accessible to antigen-specific antibodies. Bivalent VLPs induced IgG responses against both NANP and *Pf*SPECT-1 epitopes, confirming *in vivo* co-display of the target antigens. One of the bivalent candidates (N4) provided similar efficacy to R21 in formulation with Matrix-M adjuvant in *BALB/c* mice, whereas addition of *Pf*SPECT-1 significantly reduced efficacy of another candidate (N2). NANP-specific IgG response negatively correlated with blood stage parasite load. When N4 was formulated with LMQ or SMNP adjuvants, measured humoral responses or protective efficacy did not improve. Further investigation is needed to determine whether the immune response persists over time.

**Discussion:**

Our findings show that *Pf*SPECT-1 can be effectively incorporated into the HBsAg VLPs and co-displayed with CSP epitopes without impairing immunogenicity. Although protection was evaluated using transgenic *P. berghei* parasites expressing *P. falciparum* antigens, the modular HBsAg VLP platform theoretically allows incorporation of antigens from other *Plasmodium* species such as *P. vivax*.

## 1 Introduction

Malaria remains one of the most devastating infectious diseases, with *Plasmodium falciparum* (*Pf*) responsible for the majority of severe cases and deaths (1). Infection begins when sporozoites are transmitted during a mosquito bite and migrate from the skin to the liver, where they invade hepatocytes and initiate the clinically silent pre-erythrocytic stage (2). Blocking this initial phase of infection has long been recognized as a promising vaccination strategy, as it could prevent the onset of blood-stage disease and transmission (3). The *Plasmodium* evades the host’s immune response by residing primarily within host cells, making antigens from the extracellular migratory forms of the parasite’s life cycle key vaccine candidates. These antigens are exposed when the parasite is briefly outside host cells, moving through intercellular spaces or the bloodstream before invading new cells. The CSP (4, 5) and the TRAP (6) have been extensively studied sporozoite proteins to date, which play crucial roles in the traversal of mammalian cells and the invasion of hepatocytes by sporozoites. Additionally, SPECT-1 and SPECT-2 play vital roles in sporozoite movement through the dermis, sinusoidal cell layer, and Kupffer cells (5, 7, 8). These proteins, located within the sporozoite micronemes, facilitate motility and interaction with host receptors, inducing immune responses (9).

Targeted disruption of SPECT-1 has been shown to impair sporozoite infectivity *in vivo* and block traversal of HeLa cells *in vitro*, which demonstrates its critical role in crossing cellular barriers during the sporozoite’s journey from the dermis to hepatocytes (10). *In vitro* cell invasion assays have demonstrated that sporozoites lacking the *spect* gene cannot traverse Kupffer cells but retain the ability to infect hepatocytes, indicating that cell passage and infectivity are distinct processes (7, 11). Furthermore, it has been suggested that passage through Kupffer cells is not a prerequisite for hepatocyte infection (7, 8, 10). Moreover, disruption of SPECT has been shown to not affect parasite proliferation in rat erythrocytes or parasite development within the mosquito midgut or salivary glands, although it significantly impairs the parasite’s ability to infect the liver (4, 5, 7, 8, 12, 13). The findings of Patarroyo et al. (9), along with previous research on CSP by Herrera et al. (14), suggest that SPECT-1, SPECT-2, and CSP likely interact with similar host cell surface receptors to mediate cell traversal. The identification of specific sequences within the SPECT-1 protein that binds to HeLa cells, most of which exhibit high affinity for heparin and chondroitin sulphate-containing receptors, similar to other sporozoite proteins involved in cell traversal, further highlights the potential of SPECT-1 as a promising candidate for inclusion in a multi-antigenic malaria vaccine (9). Targeting its role in cell traversal, we selected SPECT-1 to be included in further modifications of current CSP-based VLP vaccine.

Both malaria vaccines, RTS, S and R21, have shown encouraging efficacy in human trials (15, 16). However, these vaccines require additional booster doses to maintain protection due to a decline in antibody titers over time (16). This underlines the critical need for a vaccine with not only high initial efficacy but also extended durability.

Efforts to develop multivalent vaccines have already seen notable success, particularly in creating VLP vaccines in both clinical and pre-clinical settings. Some multivalent VLP vaccines are already approved and widely used in clinical practice. Eminent examples include Gardasil (17) and Gardasil 9 (18), which protect against four and nine types of human papillomavirus (HPV) respectively. This approach demonstrates that using multiple antigens in the same vaccine can broaden immune protection against diverse pathogen strains, providing a framework for similar strategies in malaria vaccine development. In the context of malaria, designing a multivalent VLP vaccine that targets different antigens from the same life-cycle stage of the parasite could enhance not only the breadth of immune responses but also the efficacy. In another study, it had been tried to develop multivalent/multistage malaria vaccines by incorporating antigens from different stages and/or strains of *Plasmodium* to both prevent disease and interrupt parasite transmission; however, elicited NANP-specific antibody responses were significantly lower compared to R21 (19).

Our rationale behind the vaccine designs used in this study is grounded in the critical functions of CSP and SPECT-1 proteins in the parasite’s life cycle. While SPECT-1 contributes to the motility and cell traversal capabilities of sporozoites, facilitating their migration from the dermis to the liver (7), CSP plays a central role in the initial entry of the parasite into hepatocytes (20). By targeting both antigens within the same VLP unit, our approach aims to create a more robust and protective immune response that can simultaneously address different aspects of parasite invasion at the pre-erythrocytic stage. A C-tag was also included in the sequences to enable protein purification by affinity chromatography, as a Phase I clinical trial of a blood-stage malaria vaccine demonstrated that Good Manufacturing Practices (GMP)-grade VLPs containing a C-tag are safe in humans (NCT02927145) (21).

Based on their demonstrated immunogenicity in diverse vaccine platforms, Matrix-M, SMNP, and LMQ were selected for evaluation in our study. Matrix-M is a saponin based nanoparticle adjuvant that is composed of three primary components: saponins extracted from the bark of the *Quillaja saponaria* (Soapbark) tree, cholesterol, and phospholipids (22). Matrix-M induces robust and durable antibody and T-cell responses, promoting antigen drainage to local lymph nodes (23). It also possesses antigen dose-sparing capabilities and supports the development of a balanced Th1/Th2 CD4+ T cell immune response (24). Alongside R21 (16), it has been successfully used in licensed or late-stage vaccines against COVID-19 (Novavax) (25, 26), seasonal influenza (27), and Ebola Virus Disease (28). SMNP (Saponin/MPLA Nanoparticles) is a novel immune-stimulatory complex (ISCOM)-like combination adjuvant formed by the self-assembly of saponins with MPL-A, a TLR4 agonist. It enhances lymph flow and lymph node permeability, stimulates murine bone marrow–derived DCs to secrete cytokines (29). It has demonstrated superior humoral immunogenicity compared with aluminum hydroxide in non-human primates and has recently entered phase 1 clinical evaluation (NCT06033209) (30) and been manufactured at GMP-grade (31). LMQ is a liposomal adjuvant combining a synthetic TLR4 agonist with the saponin QS-21 that promotes Th1-biased immune responses (32). It has been tested across multiple vaccine platforms and shown properties as such good efficacy and high neutralizing antibody titers in MERS and SARS-CoV-2 studies (33, 34); enhanced CD4-T cell responses and provided protection against *Mycobacterium tuberculosis* (35); and when used with R21 malaria vaccine, it achieved 81-100% sterile protection in preclinical studies (36).

## 2 Materials and methods

### 2.1 Construction of monovalent and bivalent VLPs

Each gene sequence for both monovalent and bivalent VLPs was ordered from GeneArt (Thermo Fisher Scientific, USA) with several modifications. The sites for BamHI and NotI restriction enzymes, Kozak sequence, and the tissue Plasminogen Activator (tPA) leader sequence were added. For *spect1* gene, *Pf* 3D7 strain (PF3D7_1342500) was used. Constructs (N1, N2, N4, and N5) were designed to generate 4 different VLPs. The monovalent SPECT-1 VLP (N1) sequence was designed by fusing the full-length *Pf*SPECT-1 coding sequence at the 5′ end, followed by sHBsAg and a C-tag at the 3′ end. For the bivalent candidates, *Pf*SPECT-1 was incorporated in different configurations with CSP-derived sequences. In construct N2, the SPECT-1 sequence was inserted immediately downstream of the 18 NANP repeats and C-terminal residues of *Pf*CSP, which were retained in the same configuration as in the R21 vaccine. Construct N4 was designed with SPECT-1 fused to the 3′ end of the R21 monomeric subunit, immediately upstream of the C-tag. Finally, construct N5 was designed with SPECT-1 positioned at the 5′ end and the NANP repeats placed toward the 3′ end immediately upstream of the C-tag. For all constructs, the pre-S2 domain of hepatitis B virus (PVTN) was used as a spacer sequence directly upstream of HBsAg and was also included downstream of sHBsAg in constructs N4 and N5 as shown in **Table 1**.

**Table 1:** Monovalent and bivalent VLPs consisting SPECT-1 and/or CSP epitopes.

Short name	Construction	Size (kDa)	Size (bp)
N-1	SPECT-1+ PreS2 HepB + HBsAg + C-tag	54.72	1456
N-2	(18 NANP + C-term of CSP) + SPECT-1+ PreS2 HepB + HBsAg + C-tag	73.81	1996
N-4	(18 NANP + C-term of CSP) + PreS2 HepB + HBsAg + PreS2 HepB + SPECT-1 + C-tag	74.22	2008
N-5	SPECT-1 + PreS2 HepB + HBsAg + PreS2 HepB + (18 NANP + C-term of CSP) + C-tag	74.22	2008

C‑term, carboxyl terminus; CSP, circumsporozoite protein; HBsAg, hepatitis B surface antigen; PreS2 HepB, hepatitis B virus PreS2 region linker (PVTN); SPECT‑1, sporozoite protein essential for cell traversal‑1.

### 2.2 Cloning and transformation

The GeneArt plasmid was digested to break the plasmid backbone using restriction enzymes, BamHI, and NotI with additional EcoRI to stop plasmid re-ligation. The digested product was ligated using the same restriction enzymes, NotI and BamHI, into a linearized pPIC3.5K vector (9004 bp). The plasmid was linearized by the restriction enzyme SalI and extracted using midi-prep after transformation into *E. coli* and amplification. SalI linearizes the plasmid at the His4 site and facilitates insertion in the *P. pastoris* (GS115) genome. Since GS115 is an auxotroph that requires histidine, only transformed clones can grow in media lacking histidine. Electroporation was used to transform competent GS115 cells, which were then plated on Minimal Dextrose (MD) agar and incubated at 28 ˚C for small-scale culture growth intended for expression investigation, positive colonies were selected after three to five days.

### 2.3 Screening of clones and selection for expansion

Isolated colonies were picked up from each plate and inoculated into a buffered glycerol-complex medium (BMGY). Protein production was induced under alcohol oxidase-1 (AOX1) promoter by transferring cultures from glycerol-containing BMGY medium to buffered methanol-complex medium (BMMY), 48 hours after growth initiation. The cultures were then induced for another 48 hours with methanol feed every 12 hours. Protein expression was confirmed by Western Blot analysis using anti-NANP, anti-HBsAg, anti-C-tag primary antibodies, and polyclonal serum of *Pf*SPECT-1. The clones confirmed positive on relevant blots were chosen for expansion.

For expansion, the culture was initiated by picking a portion of the confirmed clone colony from the plate and inoculating it into BMGY in a 250 mL shaker flask. After 24–48 hours, 10 mL of culture was transferred to a 2.5 L flask with 1 L BMGY and grown for 2–3 days. The culture was induced when the OD600 reached 10–15 absorbance units. For induction, the culture was centrifuged, and the pellet was re-suspended in BMMY. The cultures were fed with methanol starting from 0.5% increasing up to 2% (v/v), harvested 3 days post-induction and the pellets were stored at -80 °C until purification.

### 2.4 Purification of VLPs

The pellets were thawed at room temperature (RT), resuspended in lysis buffer (20 mM Tris pH:8, 5 mM EDTA), and lysed in a high-pressure homogenizer (LM20 Microfluidizer^®^ Processor) (~1200 bar). The lysed material was clarified to remove debris and homogenized again in the liberation buffer (20 mM Tris pH:8, 5 mM EDTA, 0.5% Tween-20) to release VLPs. The clarified material filtered with 0.8 μm followed by a 0.22 μm filter. The filtered material was loaded onto the affinity C-tag column (20 ml of CaptureSelect™ C-tag Affinity Matrix (Thermo Scientific, 1943072250) was packed into an empty XK 16/20 column (GE Healthcare Life Sciences, 28-9889-37)). The protein was eluted from the column into fractions using 2 M MgCl_2_. The eluted affinity column fractions were pooled, filtered through 0.22 μm filter and loaded (~ 10mL) onto HiLoad^®^ 16/600 Superdex^®^ 200 pg SEC column (GE Healthcare Life Sciences, Cytiva, 28-9893-35) for buffer exchange into Tris buffered saline (TBS) (150 mM NaCl, 20 mM Tris) and collected as fractions. The fractions were pooled after being analyzed using SDS-PAGE and western blot to confirm the presence of the protein. All affinity chromatography and size exclusion chromatography (SEC) were performed using an Äkta Pure Chromatography system (GE Healthcare Life Sciences).

### 2.5 Confirmation of protein expression and VLP formation

Protein samples for the SDS-PAGE were prepared in 4 x NuPAGE™ LDS sample buffer (Invitrogen, NP0007) and 10 x NuPAGE™ sample reducing agent (Invitrogen, NP0009) and incubated at 100 ˚C for 15 minutes prior to loading. Positive control (R21) and samples (N1, N2, and N4) were loaded at 1.5 µg per well, and 0.5 µg per well used for N5. The samples and positive control were run on NuPAGE™ 8% Bis-Tris Midi Gel (Invitrogen, WG1002) using NuPAGE™ MES SDS Running Buffer (Invitrogen, NP0002-02). Protein in the gel was visualized using Coomassie staining (Quick Coomassie Stain, Neo Biotech, NB-45-00078) by dying the gel for 15 minutes then washing for 2 hours with distilled water as per kit instructions. Western blotting was performed using Transblot Turbo Transfer System (Bio-Rad, 1704150) on 0.2 μm nitrocellulose membrane (Bio-Rad, 1704159) and blocking the membrane with Blocker (Casein in PBS, Thermo Scientific, 37528) for one hour. The membrane was incubated in relevant primary antibodies (anti-NANP (in-house), anti-SPECT-1 (in-house, polyclonal), anti-HepB (Genetex, GTX40707), or anti-C- tag (Thermo Scientific, 7103252100) for two hours. After washing the membrane three times with PBS/T and one time with PBS, the membranes were incubated in anti-mouse IgG with alkaline phosphatase secondary antibody (Jackson ImmunoResearch, 715-055-150) for one hour and then washed again before developing. One BCIP/NBT tablet (Sigma, B5655) was dissolved in 10 mL distilled water to develop the membranes. The membranes were washed in distilled water after the development of bands.

Protein quantification was performed using a Pierce BCA kit (Thermo Scientific, catalogue no. 23227) according to the manufacturer’s microplate protocol. Briefly, 25 μL of each protein sample or BSA standard was added to a 96-well plate, followed by 200 μL of BCA working reagent (prepared by mixing Reagents A and B in a 50:1 ratio). The plate was incubated at 37 °C for 30 minutes, then cooled to room temperature. Absorbance was measured at 562 nm using CLARIOStar Plus (Isogen Life Sciences, BMG LABTECH). Protein concentrations were calculated based on a standard curve generated from serial dilutions of bovine serum albumin (BSA).

VLPs were subjected to negative staining by incubating 10 ul of sample on glow-discharged carbon grids (TAAB, C267/050) for two minutes and then with 2% uranyl acetate for 10 seconds after blotting on filter paper. Transmission electron microscopy was performed using a JEOL-1400 microscope operated at 120 kV to analyze the shape and the integrity of the VLPs.

### 2.6 *In vitro* confirmation of epitope display

Binding of antigen-specific antibodies to VLP-coated wells was used to assess the display of epitope accessibility and antigen display *in vitro* via ELISA using Nunc-Immuno MaxiSorp 96-well plates (Thermo Scientific, 442404). Plates were coated overnight at 4 °C with purified VLPs diluted in carbonate–bicarbonate coating buffer (Sigma-Aldrich, C3041) at a concentration of 2 µg/mL. Plates were washed with PBS containing 0.05% Tween-20 (PBS-T) and blocked with Casein in PBS (Thermo Scientific, 37528). Following blocking, plates were incubated with either the monoclonal anti-NANP antibody 2A10 or polyclonal anti-SPECT-1 antibodies in triplicates, starting at a 1:200 dilution followed by three-fold serial dilutions (three steps). Antibodies were incubated for two hours at room temperature. Plates were then washed with PBS-T and incubated for one hour at room temperature with alkaline phosphatase–conjugated anti-mouse IgG (Sigma-Aldrich, A3562). After a final wash, plates were developed using p-nitrophenyl phosphate substrate (Sigma-Aldrich, N2765) at 1 mg/mL in diethanolamine buffer (Sigma-Aldrich, 34064) for 15 minutes. Absorbance was measured at 405 nm.

### 2.7 Viral vector vaccines

Chimpanzee adenovirus (ChAd63) and Modified Vaccinia Ankara (MVA) encoding SPECT-1 were kindly provided by Ahmed M Salman and produced by AMS and NKA.

### 2.8 Adjuvants

Matrix-M adjuvant, lot M1-118 (0.75 mL, 0.375 mg/mL) has been provided by Novavax under an MTA to be used in the vaccination studies. Saponin/MPLA nanoparticles (SMNP) adjuvant has been provided by Massachusetts Institute of Technology (MIT) under an MTA to be used in the vaccination studies. LMQ adjuvant was manufactured at the Vaccine Formulation Institute (VFI, Switzerland) and provided under an MTA to be used in the vaccination studies.

### 2.9 Animals

All animal experiments and procedures were conducted under the UK Animals (Scientific Procedures) Act 1986 and approved by the University of Oxford Animal Care and Ethical Review Committee for use. All animal experiments were conducted in accordance with the 3Rs principles. While *in vitro* methods were used wherever possible, the use of animals in challenge experiments was essential and could not be fully replaced, as no alternative models currently replicate the complexity of *P. falciparum* infection and protective immunity. Power calculations were used for determining the smallest number of animals needed per group to detect meaningful effects for reduction principle. Refinement was ensured through humane handling, regular monitoring, and minimization of discomfort throughout the study. Animals were grouped and housed in individually ventilated cages under specific pathogen-free conditions, with constant temperature, humidity and a 12:12 light-dark cycle (8 am to 8 pm). For induction of short-term anesthesia, animals were either injected with IM (intramuscular) xylazine (~7 mg/kg) and ketamine (~70 mg/kg) or anaesthetized using vaporized IsoFlo^®^ (3.5%, 2 liter/minute oxygen). All animals were humanely sacrificed at the end of each experiment by an approved Schedule 1 method (neck dislocation). All efforts were made to minimize suffering.

### 2.10 Immunization

Female *BALB/c* mice (6 weeks old) were purchased from Inotiv (formerly Envigo) and were housed in specific pathogen-free environments. The mice were vaccinated under isoflurane-induced general anesthesia by intramuscular injection of 50 μl vaccine formulation into one leg. For all VLP vaccinations, 1 μg of VLP was mixed with 5 μg adjuvant Matrix-M (MM) or 5 μg adjuvant SMNP or 2 μg of TLR-4 agonist 3D6AP and 5 μg of QS21 saponin (each injectable dose of LMQ adjuvant). For viral vector vaccinations, 1x10^8^ infectious-forming units (ifu) of ChAd63 and 1x10^7^ plaque-forming units (pfu) of MVA encoding SPECT-1 were used per mouse per injection. The volumes were made up with sterile PBS when required. Vaccination was performed within one hour of formulation preparation which was kept on ice until injected. Prime vaccination was followed by the booster with three weeks interval. Blood samples were collected at two time points; two days before boost vaccination and challenge. Blood samples were allowed to clot by storing it at 4 ˚C overnight before centrifuging it at 13000 rpm for 10 minutes to separate the sera. Then sera were stored at -20 °C until use.

### 2.11 Challenge

For parasite maintenance, frozen *P. berghei* pRBCs (parasitized Red Blood Cells) from the chimeric parasite line PfSPECT1(r)PbSPECT1+PfCSP(r)PbCSP (2702 cl1) were thawed and 100–300 μl of infected whole blood was injected intraperitoneally (IP) into a naïve *BALB/c* mouse. *BALB/c* mice have approximately 8-10 × 10^6^ RBCs per μl; thus, the inoculum contained ~8 × 10^8^ to 3 × 10^9^ total RBCs, of which ~5-6% (approximately 4 × 10^7^ to 1.8 × 10^8^) were parasitized, depending on donor parasitemia. After five to six days, parasitemia was monitored by microscopic examination of Giemsa-stained thin blood smears. Gametocytemia was not specifically quantified; however, representative images of gametocytes from the transgenic *P. berghei* parasite observed during the challenge study are shown in **Supplementary Figure 3**. Anaesthetized mice with approximately 5-6% parasitemia were then exposed to pots containing starved female *Anopheles stephensi* mosquitoes (four to seven days old) for 10–15 minutes. Infected mosquitoes were kept at 19–23 °C in a humidified incubator and fed with the fructose solution. At 21–23 days of post-feeding, mosquito salivary glands were dissected to collect infectious sporozoites in suitable cell culture media, followed by homogenization on ice. Sporozoite numbers were determined using a hemocytometer under a phase-contrast microscope. To evaluate vaccine efficacy, vaccinated and naïve mice were challenged with 1,000 sporozoites via intravenous (IV) injection into the lateral tail vein approximately three weeks after the second (boost) dose. Mice were monitored from day five after the challenge using thin blood smears. Animals with parasite-positive smears on three consecutive days were sacrificed. Parasitemia from three smears was used to calculate time to 1% parasitemia by linear regression. If smears remained negative at 14 days of post-infection, mice were considered protected and sacrificed.

### 2.12 Thin blood films

Thin blood smears were prepared on glass slides from a drop of blood obtained from the tail snip. The blood smear was allowed to air-dry, then fixed with methanol, and stained for 0.5–1 h with 5% Giemsa diluted in Milli-QH2O. Slides were then air dried at room temperature and viewed under a light microscope at 100x under oil immersion. To predict the time to 1% blood-stage infection, a linear regression modelling was used as described previously (37). The logarithm to base 10 of the calculated percentage of parasitemia was plotted against the time post-challenge and GraphPad Prism 10 statistical analysis software used for generating a linear regression model on the linear part of the blood-stage growth curve.

### 2.13 Anti- NANP Standard ELISA

ELISAs were carried out in Nunc-Immuno Maxisorp 96 well plates (Thermo Scientific, 442404) coated with 2 μg/ml of (NANP)6C in carbonate-bicarbonate coating buffer (Sigma Aldrich, C3041) overnight at 4 °C. Plates were washed with PBS-Tween and blocked with Casein in PBS (Thermo Scientific, 37528). Sera were diluted to reach an OD405 in the linear range of the standard curve at the same time an internal control reached an OD405 of 1. Samples were analyzed in triplicates. Anti-NANP mAb (2A10) was used to build standard curve. Plates were incubated for two hours at room temperature and then washed as before. The anti-mouse whole IgG conjugated to alkaline phosphatase (AP) (Sigma, A3562) was added as a secondary antibody and then incubated for one hour at room temperature. Following a final wash, plates were developed by adding p-nitrophenyl phosphate (Sigma, N2765) at 1 mg/mL in diethanolamine buffer (Sigma, 34064) and read at 405 nm. ELISA units were extrapolated from the samples’ OD on the linear range of the standard curve. Samples with OD (optical density) values <0.1 were assigned a titer of 100 (dotted line) and considered non-responders.

### 2.14 Endpoint ELISA

Antibody responses to SPECT-1 in mouse sera were assessed by endpoint IgG ELISA. ELISAs were carried out in Nunc-Immuno Maxisorp 96 well plates (Thermo Scientific, 442404) coated with 2 μg/ml of the purified SPECT-1 in carbonate-bicarbonate coating buffer (Sigma Aldrich, C3041) overnight at 4 °C. Plates were washed with PBS-Tween and blocked with Casein in PBS (Thermo Scientific, 37528). Diluted sera were added to the top row of the plate in duplicate and serially diluted three-fold down the plate. Plates were incubated for two hours at room temperature and then washed as before. Anti-mouse whole IgG-AP (Sigma, A3562) was added for one hour at room temperature. Following a final wash, plates were developed by adding p-nitrophenyl phosphate (Sigma, N2765) at 1 mg/mL in diethanolamine buffer (Sigma, 34064) and read at 405 nm. Serum antibody endpoint titers were taken as the x-axis intercept of the dilution curve at an absorbance value of the background plus three standard deviations or a minimum of 0.15. If a 1:100 dilution of a serum sample did not develop a signal above the background, the IgG titer in this sample was considered to be 0. To make comparable results, a serum pool was included on each plate as an internal control.

### 2.15 Anti- NANP avidity ELISA

The avidity of anti-NANP antibodies was determined by chaotropic salt displacement ELISA. Serum samples were diluted to the dilution at which the OD405 in the Standard ELISA had been 1. Diluted sera were then added to two columns of a Nunc-Immuno Maxisorp 96 well plates (Thermo Scientific, 442404) that had been coated with 2 μg/ml (NANP)6C peptide in carbonate-bicarbonate coating buffer (Sigma Aldrich, C3041) overnight at 4 °C. Plates were incubated for two hours at room temperature and then washed 6 times in PBS-T. Increasing concentrations of sodium thiocyanate (NaSCN) diluted in PBS were added down the plate (0, 0.5, 1, 1.5, 2, 2.5, 3, and 3.5M). Plates were incubated with the chaotropic salt for 15 minutes at room temperature before plates were washed again six times in PBS-T. Goat anti-mouse whole IgG conjugated to alkaline phosphatase (Sigma, A3562) was added for one hour at room temperature. Following a final wash as mentioned above, plates were developed by adding p-nitrophenyl phosphate (Sigma, N2765) at 1 mg/mL in diethanolamine buffer (Sigma, 34064) and read at 405 nm. Avidity was calculated as the IC50 of NaSCN, i.e., the concentration of NaSCN at which the signal is exactly half the intensity of the signal when no NaSCN was added.

### 2.16 Statistical analysis

Statistical analysis was performed using GraphPad Prism version 10 (GraphPad, USA) unless indicated otherwise. Power calculations were conducted for both antibody magnitude (one-way ANOVA) and time to 1% parasitemia (log-rank test). With 6-8 mice per group across two or more groups, the study had ≥80% power to detect large effect sizes (f ≥ 0.4 for antibody titers, hazard ratio ≥ 3 for time to parasitemia) was not powered to detect modest differences in sterile protection between groups. Correlation analyses were powered to detect moderate-to-strong associations (ρ ≥ 0.6). Before statistical analysis to compare two or more populations, the Kolmogorov-Smirnov test for normality was used to determine whether the values followed a Gaussian distribution. When comparing two groups, the Mann-Whitney test was used for non-parametric data, and the unpaired t-test was used for parametric data. Two or more groups of parametric data were compared by One-way ANOVA with Bonferroni’s multiple comparison test (comparing all pairs of groups) or with Dunnett’s multiple comparison test (comparing all groups to one group). Two or more groups of non-parametric data were compared with Kruskal-Wallis with Dunn’s multiple comparison test, and correlations were assessed using Spearman’s rank correlation. Challenge results are presented in the Kaplan-Meier survival graphs and survival curves were compared by Log-rank (Mantel-Cox) test. Significance was indicated when the value of p < 0.05 (*p < 0.05, **p < 0.01, ***p < 0.001).

## 3 Results

### 3.1 SPECT-1 can be displayed as an additional antigen on R21

**Figure 1:**
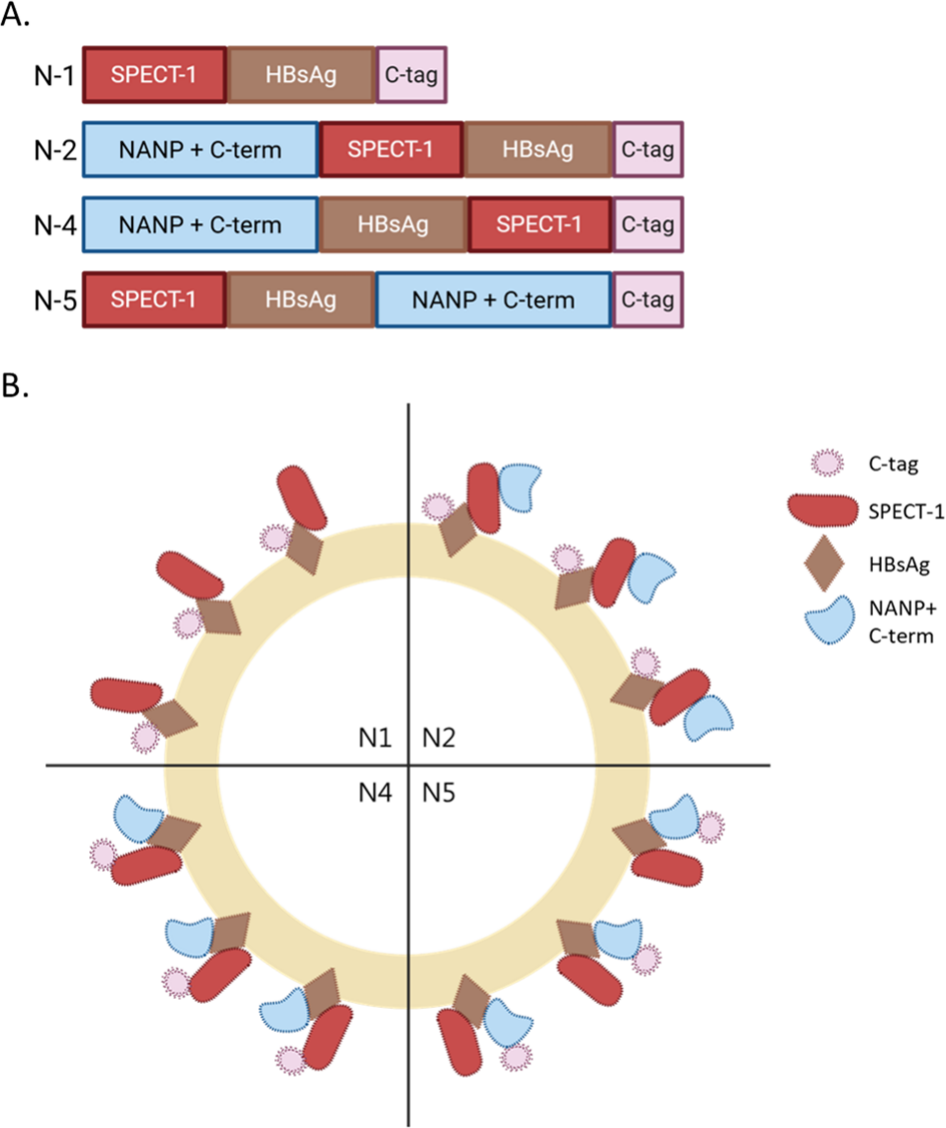
Comparison of generated monovalent and bivalent VLPs. **(A)** Schematic representation of the VLP sequences. **(B)** Proposed organization of components (SPECT-1, NANP + C term, C-tag, and HBsAg) on lipid layer. It does not represent true three-dimensional structures. Figures created via BioRender.com.

The monovalent SPECT-1 VLP (N1) sequence was designed by fusing the whole sequence of PfSPECT-1, HBsAg, and C-tag. Then for the bivalent candidates; SPECT-1 was fused to R21 sequence in various ways. For N2, it was added right after 18 NANP repeats, and C-term residues of PfCSP (R21). N4 was designed in a way that SPECT-1 fused to the C-terminus of the monomeric subunit of R21, which had been shown to be susceptible to modification by expression of the C-tag previously. Finally, N5 was designed to have SPECT-1 on the N terminus and the NANP repeats on the C terminus right before the C-tag. Pre-S2 domain of Hepatitis B virus (PVTN) was used as a spacer right before HBsAg for all constructs and right after HBsAg for N4 and N5. The orders of all candidates are as shown in **Figure 1A** and **Table 1**. The structural arrangement of the individual components within the VLP is schematically represented in **Figure 1B**.

**Figure 2:**
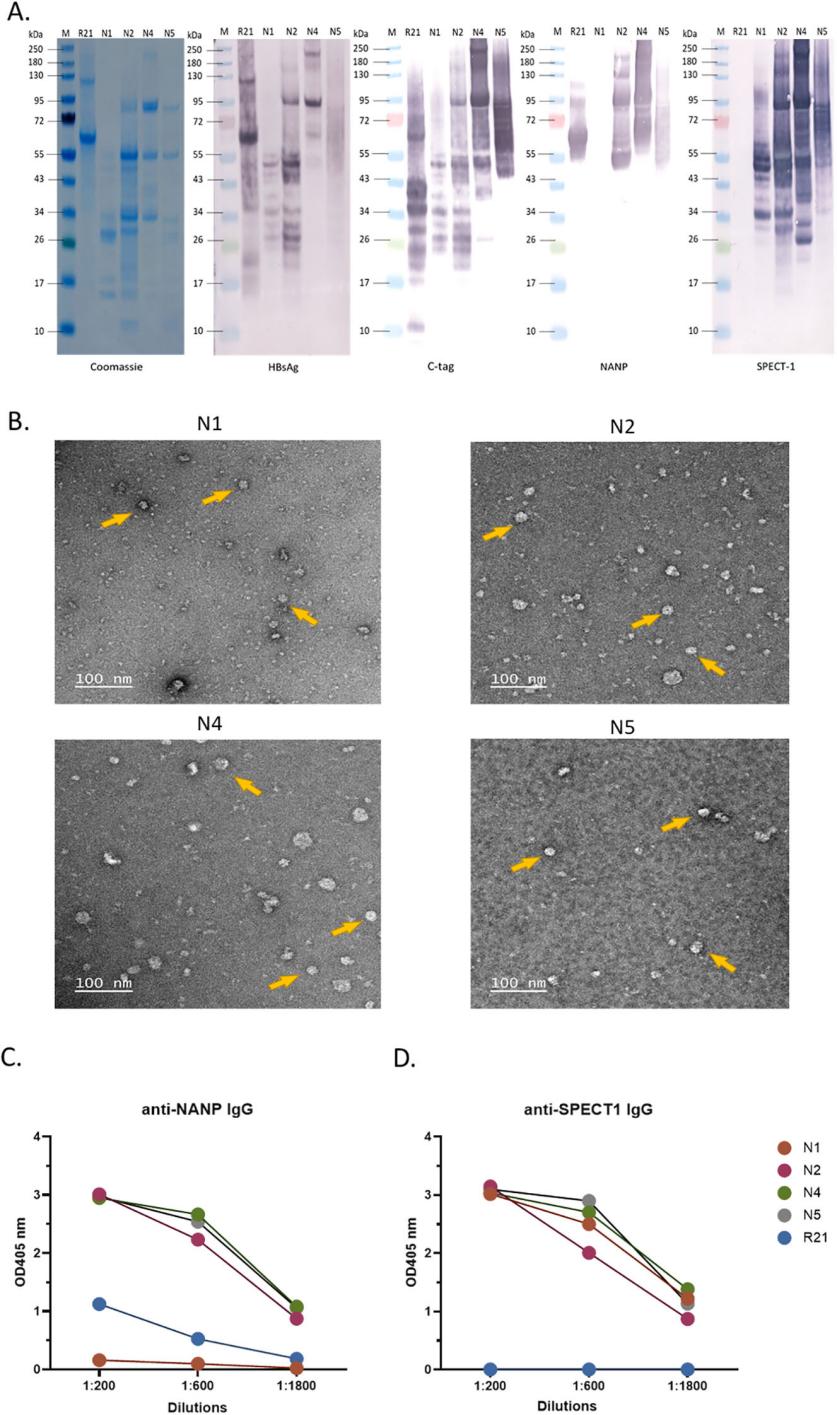
SDS-PAGE, western blot and negative staining images of the purified VLPs. **(A)** SDS-PAGE and western-blot imaging. **(B)** VLPs were subjected to negative staining with 2% uranyl acetate. Transmission electron microscopy was performed using a JEOL-1400 microscope operated at 120 kV to analyze the shape and the integrity of the VLPs. Bar = 100 nm. Antigen accessibility on VLP surfaces assessed by ELISA. Purified VLPs were used as coating antigens in a direct ELISA and incubated with **(C)** 2A10 (anti-NANP mAb) or **(D)** polyclonal anti-SPECT-1 antibodies, starting at a 1:200 dilution followed by three-fold serial dilutions.

VLPs were expressed using the *Pichia pastoris* expression system and purified via c-tag affinity chromatography followed by size-exclusion chromatography (**Supplementary Figure 1**). Particles’ integrity was confirmed by biochemical and ultrastructural analyses (**Figures 2A, B**). SDS-PAGE analysis revealed the prominent bands at approximately 55, 80, 75, 80 kDa, corresponding to the expected molecular weight of N1, N2, N4, and N5, respectively. Bands were also detected by western blot, confirming antigen expression within the VLPs (**Figure 2A**). Electron microscopy further demonstrated the presence of well-formed, spherical VLPs with an approximate diameter of 20–30 nm, consistent with the expected size of assembled particles. Representative particles are indicated by arrows in **Figure 2B**.

Purified VLPs retained antigenic integrity, as demonstrated by their ability to bind antigen-specific antibodies shown by ELISA (**Figures 2C, D**). Robust, dilution-dependent reactivity was observed with the monoclonal anti-NANP antibody 2A10 as well as with polyclonal anti-SPECT-1 which indicates that the VLPs display epitopes from both antigens are accessible and recognized by antigen-specific antibodies *in vitro*. The VLP production workflow is summarized in **Supplementary Figure 2**.

### 3.2 Monovalent and bivalent VLPs elicit robust antigen specific immune response

**Figure 3:**
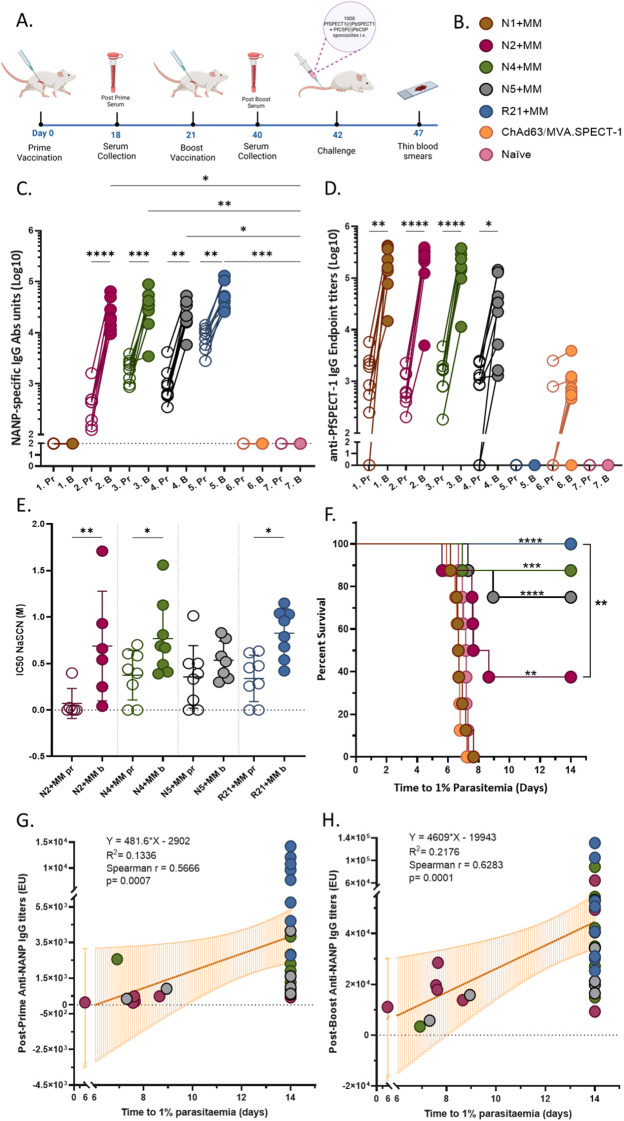
Monovalent and bivalent vaccine candidates provided immunogenicity. **(A)** Timeline for the experiment. 6-weeks old female BALB/c (n=8) were vaccinated intramuscularly with 1 μg of VLP + 5 μg of Matrix-M for prime and boost or 1x10^8^ ifu of ChAd63.SPECT-1 for prime and 1x10^7^ pfu of MVA.SPECT-1 for boost were used per mouse. Serum collection was conducted via tail bleeding 2–3 days before boost vaccination and challenge. For challenge, 1000 transgenic sporozoites were injected intravenously. Created via BioRender.com. **(B)** Figure legend for vaccination groups. **(C)** NANP specific IgG response after prime-Pr and boost-B vaccination. Prime samples are represented by open circles, while boost samples are represented by filled circles. For the samples which have less than OD value of 0.1, a titer of 100 was assigned for statistical analysis purposes. These samples are displayed on the dotted line and considered non-responders. Statistical analysis for boost vs naïve comparisons was performed using Kruskal-Wallis ANOVA with Dunn’s multiple comparisons. Prime vs boost comparisons were run separately for each group and paired t test was used for parametric results while Wilcoxon test was used for non-parametric data. **(D)** SPECT-1 specific IgG response after prime-Pr and boost-B vaccination. Prime samples are represented by open circles, while boost samples are represented by filled circles. Statistical analysis was performed using mixed-effects analysis with Šídák’s multiple comparisons test (ChAd63 = Chimpanzee Adenovirus 63 Viral Vector, MVA = Modified Vaccinia Ankara). **(E)** Avidity of NANP specific IgG. Prime samples are represented by open circles, while boost samples are represented by filled circles. Mean with SD. Statistical analysis was performed using mixed-effects analysis with Holm-Šídák’s multiple comparisons test, with single pooled variance. **(F)** Survival analysis via Kaplan Meier. Statistical significance was assessed using the Log-rank (Mantel–Cox) test. **(G)** Post-prime; **(H)** Post-boost anti-NANP IgG titers show correlation with time to parasitemia. All three groups of bivalent and the R21 group samples were combined for the analysis. Animals that remained parasitemia-free throughout the study were assigned the maximum follow-up time (day 14). The orange line indicates the linear regression model with 95% confidence interval shaded in orange. The R^2^ value for each model is displayed on the graph. Correlation analysis was performed using Spearman’s method. A value of p=0.05 or less was considered statistically significant for all analyses and displayed with asterisks (*=p≤ 0.05, **=p≤ 0.01, ***=p≤ 0.001, and ****=p≤ 0.0001). Created via BioRender.com.

A prime-boost-challenge regimen with three-week intervals was followed for each vaccinated group (**Figure 3A**). All VLPs tested alongside R21 as a control for NANP response (**Figure 3B**). A group of ChAd63/MVA.SPECT-1 was used as control for SPECT-1 response (**Figure 3B**). After prime vaccination, all NANP-employing-VLP vaccines generated NANP-specific IgG response at various levels. N2 induced low but detectable post-prime antibody levels which were not significantly different from the naïve control group (p = 0.5113). Both N4, and N5 induced significantly higher antibody levels than the naïve group (p = 0.0025 and p = 0.0348, respectively), and R21 elicited a robust response (p < 0.0001). All NANP-employing-VLP vaccines induced higher IgG levels compared to the naïve group after the boost dose. There was no statistically significant difference between the VLP vaccinated groups after boost (N2, N4, N5, and R21) (p>0.05). The boost vaccination generated a significantly higher immune response against NANP than prime in N2, N4, N5, and R21 (p=0.0043; 0.0004; 0.0149; 0.0019, respectively) None of the bivalent VLPs showed an antagonistic effect on the IgG antibody response against NANP after the boost. As expected, N1 and ChAd63/MVA.SPECT-1 groups showed only baseline activity after prime and boost vaccination, same as negative control group. (**Figure 3C**).

SPECT-1-specific IgG response was measured to capture the immune response to the monovalent VLP (N1) and the second antigen of bivalent VLPs via endpoint ELISA. After the prime vaccination, all VLP groups displaying SPECT-1 on the surface (N1, N2, N4, and N5) showed significantly higher response than naïve samples (comparison not shown on the graph, 0.0043, 0.0208, 0.0013, 0.0198, respectively). Some samples of N5 and ChAd63/MVA.SPECT-1 groups did not reach the endpoint titer. Following the boost vaccination, N1, N2, N4, and N5, successfully elicited SPECT-1-specific IgG response and showed statistically significant results compared to naïve samples (comparison not shown, <0.0001, <0.0001, <0.0001, and 0.0096, respectively). Even though all samples of N5 showed improvement, one sample of ChAd63/MVA.SPECT-1 did not show antibody response. As expected, R21 showed only baseline activity of SPECT-1-specific IgG after prime and boost vaccination (**Figure 3D**).

To assess the quality of the antibody response, avidity measurements (IC50) of NANP-specific IgG were performed. All four vaccine groups that displays NANP repeats on the surface generally exhibited an upward trend in NANP-specific IgG avidity from the prime to the boost samples (**Figure 3E**). The changes within each group, except for the N5, reached statistical significance (p=0.2969). N2, N4, and R21 showed a statistically significant increase between prime and boost samples, with a p-value of 0.0077, 0.0389, and 0.0134, respectively. These results indicated a more pronounced enhancement in antibody avidity post-boost in N2, N4, and R21. There was no statistical significance between groups in fold change results of anti-NANP avidity.

### 3.3 Bivalent VLPs are protective against IV transgenic sporozoite challenge

The challenge experiment results revealed some differences in vaccine efficacy (**Figure 3F**). Among the tested groups, R21 demonstrated the highest efficacy, achieving 100% protection by completely preventing the onset of parasitemia in all subjects (p=<0.0001). N4 and N5 vaccine candidates also showed high efficacy rates of 87.5% (p=0.0009) and 75% (p=<0.0001), respectively, in parasitemia development compared to the naïve group. N2 exhibited moderate efficacy at 37.5%, demonstrating some protective effect compared to the naïve group but less robust than the other candidates (p=0.0011). In contrast, N1 and ChAd63/MVA.SPECT-1 groups failed to provide protection, with efficacy rates of 0%, showing no measurable delay in parasitemia compared to the naïve group. All groups compared with each other individually using the same test and no significance was detected except R21 protected significantly better than N2 (p=0.0085).

Protection against *P. falciparum* infection has been correlated with specific antibody responses, particularly with IgG titers against NANP repeat region of CSP (38). IgG levels against NANP repeats from all three bivalent groups (N2, N4, and N5) and R21 were combined for correlation analysis with time to parasitemia to assess whether a general relationship existed between antibody levels and protection across the bivalent constructs. By analyzing the combined data set, the goal was to identify overall trends that were not statistically significant within each individual group due to limited sample sizes. The findings in this study reinforce this concept, as a positive correlation between IgG titers and time to parasitemia was observed in both post-prime and post-boost vaccinations (p=0.0007, 0.0001, respectively) (**Figures 3G, H**). The results indicate that higher anti-NANP IgG levels are associated with delay or protection in parasitemia in bivalent vaccines. Despite the tested vaccine constructs containing identical amounts of NANP, N2 did not follow the same pattern as the other bivalent vaccines. Unlike its counterparts, N2 failed to exhibit a correlation with protection. The correlation analysis between anti-SPECT-1 IgG antibodies and infection (time to parasitemia) suggests that these antibodies did not significantly contribute to protection against infection in the tested model (**Supplementary Figure 4**).

### 3.4 Bivalent VLPs with adjuvants Matrix-M, SMNP, and LMQ elicit antigen specific responses and contribute to protection at varied levels

**Figure 4:**
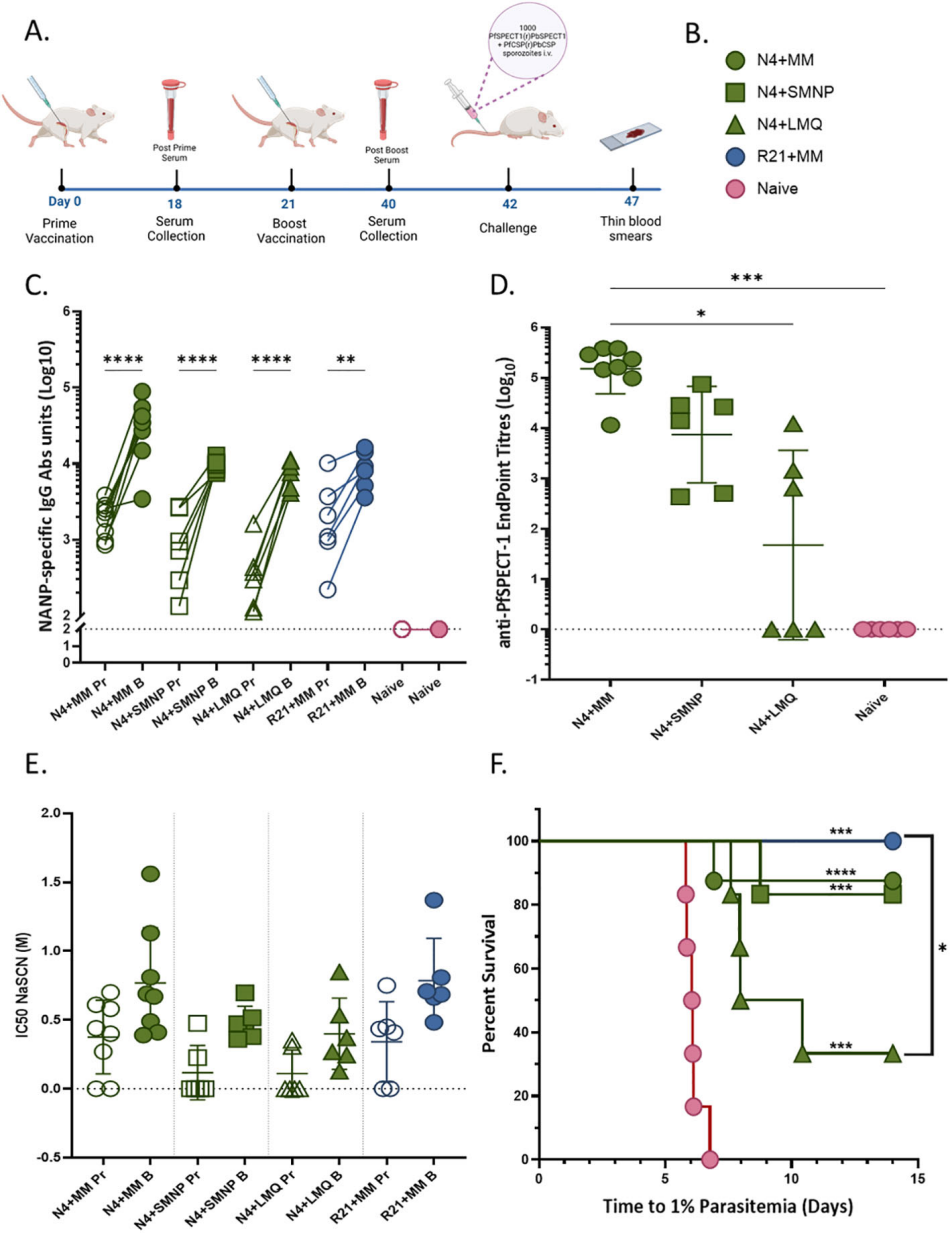
Novel adjuvants provide various levels of immunogenicity and protection. **(A)** Timeline for the experiment. 6-weeks old female *BALB/c* (n=6) were vaccinated intramuscularly with 1 μg of VLP mixed with 5 μg adjuvant Matrix-M (MM) or 5 μg adjuvant SMNP or 2 μg of TLR-4 agonist 3D6AP and 5 μg of QS21 saponin (adjuvant LMQ) for prime and boost were used per mouse. Serum collection was conducted via tail bleeding 2–3 days before boost vaccination and challenge. For challenge, 1000 transgenic sporozoites were injected intravenously. Created via BioRender.com. **(B)** Figure legend for vaccination groups. **(C)** NANP specific IgG response after prime (Pr) and boost **(B)** vaccination. Prime samples are represented by open shapes, while boost samples are represented by filled shapes. For the samples which have less than OD value of 0.1, a titer of 100 was assigned for statistical analysis purposes. These samples are displayed on the dotted line and considered non-responders. Statistical analysis for boost vs naïve comparisons was performed using Kruskal-Wallis ANOVA with Dunn’s multiple comparisons. Prime vs boost comparisons were performed using mixed-effects analysis with Holm-Šídák’s multiple comparisons test, with single pooled variance. **(D)** SPECT-1 specific IgG response after boost vaccination. Statistical analysis for SPECT-1 was performed using Kruskal-Wallis ANOVA with Dunn’s multiple comparisons test. **(E)** Avidity of NANP-specific IgG. Prime samples are represented by open circles, while boost samples are represented by filled circles. Mean with SD. Statistical analysis was performed using Kruskal-Wallis ANOVA with Dunn’s multiple comparisons. **(F)** Survival analysis via Kaplan Meier. Statistical significance was assessed using the Log-rank (Mantel–Cox) test. A value of p=0.05 or less was considered statistically significant for all analyses and displayed with asterisks (*=p≤ 0.05, **=p≤ 0.01, ***=p≤ 0.001, and ****=p≤ 0.0001). Created via BioRender.com.

Based on the performance of N4, further experiments were conducted to evaluate how adjuvant formulation influences its immunogenicity. A prime–boost vaccination regimen was administered with a three-week interval between doses, followed by a challenge with transgenic *Plasmodium berghei* parasites (**Figure 4A**). Two alternative adjuvants, SMNP and LMQ, were tested in combination with 1 μg of N4 VLP and compared to the reference R21+Matrix-M formulation. Three groups of six female *BALB/c* mice were vaccinated, while an additional group of six mice served as a negative control (**Figure 4B**). The N4+MM data were displayed as a visual comparison and it is taken from the previous experiment that is shown in the section **3.2** and **3.3** since the same vaccination plan was followed in both experiments. All adjuvanted VLP formulations successfully induced NANP-specific IgG responses after the prime immunization, which were markedly enhanced following the booster (**Figure 4C**). The booster dose was effective in every vaccinated mouse, resulting in a significant increase in antibody titers across all vaccinated groups compared to prime (*p* < 0.001 for SMNP and LMQ; *p* = 0.0007 for R21+MM). Among the groups, both SMNP and LMQ formulations generated strong NANP-specific IgG responses, comparable to those observed with the R21+MM controls. There was no statistically significant difference between MM and SMNP (p=0.5831) or between SMNP and LMQ (p=0.9913) groups in terms of SPECT-1-specific IgG antibody response after boost vaccination (**Figure 4D**). A statistically significant difference was observed between MM and LMQ (p=0.0101). When compared to naïve, MM provided a statistically significant difference (p=0.0002) in contrast to the SMNP and LMQ groups (p=0.1122, p>0.9999, respectively). The difference in avidity of NANP-specific IgG antibodies post-prime and post-boost was not significant for any groups (p=0.2428 for N4+MM, p=0.1688 for SMNP, p=0.3817 for LMQ, p=0.1156 for R21+MM (**Figure 4E**). There was also no significant difference when compared to boost samples of all groups. The efficacy of all groups after challenge varied (**Figure 3F**). While R21+MM protected all mice (p=0.0005), it was 87.5% efficacy in N4+MM group (p<0.0001). N4+SMNP protected 83.3% (p=0.0005). N4+LMQ provided 33.3% efficacy (p=0.0005).

## 4 Discussion

Efforts to develop next-generation malaria vaccines aim to improve the immunogenicity and protective efficacy of RTS,S/AS01 and the more recent R21/Matrix M. Both vaccines composed of HBsAg-based VLPs displaying NANP repeats to induce protective antibody responses (39, 40). Here, we extended this approach by designing recombinant HBsAg-based bivalent VLPs incorporating an additional *P. falciparum* antigen, SPECT-1, to explore whether multivalent antigen display could provide protective immunity.

VLP-based indirect ELISA analysis, which has been used in various studies (41–43), indicated that both CSP-derived NANP repeats and SPECT-1 on bivalent candidates were surface-exposed and accessible to antigen-specific antibodies (**Figures 2C, D**). Negative staining showed that, as previously reported, all HBsAg-based VLP constructs formed heterogenous particles which suggest variability in particle assembly (44, 45). Although the bivalent constructs were designed to display CSP and SPECT-1 at one-to-one ratio, a key limitation of this study was the inability to directly quantify antigen density or distribution on the particle surface. This is particularly relevant, as antigen density and spatial organization on nanoparticle platforms are known to influence B cell receptor crosslinking and downstream immune activation (46–48). The lack of precise structural characterisation also limits the ability to assess whether antigen competition or steric effects contribute to differences observed between constructs.

All bivalent vaccine candidates elicited antibody responses against both antigens in BALB/c mice, confirming successful antigen incorporation. Differences in avidity among constructs suggest that antigen configuration may influence the kinetics and quality of the immune response which had been also suggested in another VLP-based vaccine study (Cervarix) (49). Consistent with previous studies, we found that higher anti-NANP avidity was associated with reduced parasitemia (50, 51). Samples with lower avidity of anti-NANP IgG antibodies showed a higher likelihood of blood-stage parasitemia post-challenge. In particular, the sample with the lowest avidity also exhibiting the highest parasite count within each bivalent group (data not shown) reinforces the hypothesis that biophysical properties of vaccine-induce antibody affinity are likely to correlate with protection against *P. falciparum* challenge (52).

Challenge findings demonstrated a spectrum of protective efficacy across constructs, with R21 leading as the benchmark and N4 and N5 showing the most promise among bivalent candidates. The lack of protection observed with N1 and the lack of correlation between anti- SPECT-1 antibody titers and time to parasitemia indicate that SPECT-1 alone is insufficient to confer protection in this model, potentially reflecting both structural limitations and the nature of the immune response it induces. It also suggests that protection is primarily CSP-driven, and that the role of SPECT-1 remains unresolved. Similar observations in multivalent constructs have been reported in recent studies where the addition of alternative epitopes or antigens did not improve protection despite inducing measurable antibody responses (51).

Adjuvant selection markedly influenced protective outcomes, despite comparable NANP-specific antibody titers. Matrix-M emerges as the most effective adjuvant which highlights the superior immunostimulatory capacity in enhancing the immunogenicity and protective efficacy of the bivalent VLP vaccine. This might be attributed to its capacity to trigger antigen cross-presentation with rapid distribution from vaccination site to draining lymph nodes (24, 53).

Correlation analyses confirmed the association between anti-NANP IgG responses and protection (50). The lack of correlation observed in the N2 construct may be attributed to its unique antigen arrangement, where both antigens are positioned on the N-terminus of the sequence. In contrast, other bivalent vaccine candidates that distribute antigens across opposite sides of the VLP structure exhibited better immunogenic and protective profiles. This suggests that beyond the quantity of NANP repeats, the spatial arrangement and sequence order significantly influence the immune response and its correlation with protection (47, 48).

The lack of correlation between the levels of SPECT-1-specific IgG antibody and time to 1% parasitemia suggests that humoral responses alone may not fully account for the contribution of this antigen. This may reflect differences in antigen function, accessibility, or mechanism of action during infection. In addition, the intravenous challenge model used in this study bypasses the natural route of sporozoite migration and may underestimate the contribution of SPECT-1 (54). This model does not allow direct assessment of SPECT-1 function in sporozoite traversal. An intradermal or mosquito-bite challenge model could have produced different results by replicating the natural route of the parasite as shown by a Phase IIb study (55). Future studies employing such models could help clarify whether SPECT-1 contributes to protection under physiologically more relevant conditions and better define its potential role in multivalent vaccine strategies.

While these observations suggest that SPECT-1-associated effects may not be fully explained by humoral responses, the underlying mechanisms remain unclear. Our study primarily focused on humoral immune responses and protective efficacy. Although detailed cellular immune profiling was not performed, prior depletion studies in this model did not support a dominant role for CD8+ T cells, suggesting that alternative mechanisms, including CD4+ T cell-associated responses or antibody-mediated effects, may contribute (**Supplementary Figure 5**). Nevertheless, functional antibody assays and comprehensive cellular analyses will be required to further define the mechanisms of protection.

Our findings highlight the importance of antigen configuration in VLP-based vaccine design. While the protective role of NANP repeats is well established (51, 56), we suggest that the physical configuration of each antigen within the vaccine structure also plays a pivotal role. Variations in antigen presentation, even though the quantity of NANP repeats is constant, can lead to significant differences in immunogenicity and efficacy.

Our data indicate that incorporating a second antigen into a VLP formulation does not compromise the humoral immune response elicited by R21 alone, as the bivalent constructs exhibited comparable immunogenic profiles. However, in contrast to a recent study showing synergistic effects of combining CSP with additional antigens on nanoparticle platforms (57), the addition of SPECT-1 in this context did not enhance protection. R21 and Matrix-M formulation was the most potent and protective of the immunogens tested.

Similar approaches aimed at combining multiple antigens or targeting different parasite stages are increasingly being explored to improve malaria vaccine efficacy (57–59). Future work should focus on refining antigen presentation, optimizing VLP stability, and evaluating the functional immune responses induced by these vaccine candidates.

## Data Availability

The original contributions presented in the study are included in the article/**Supplementary Material**. Further inquiries can be directed to the corresponding authors.
