# Supplementary material for: Bivalent virus-like particles expressing SPECT1 and CSP trigger pre-erythrocytic malaria immunity and protect against transgenic Plasmodium falciparum sporozoite challenge in mice
Source: Front Immunol. 2026 May 14;17:1790309. doi: 10.3389/fimmu.2026.1790309 (PMC13216468; doi:10.3389/fimmu.2026.1790309)
Supplement: Supplementary file 1 [file DataSheet1.docx]

Supplementary Material

# Supplementary Figures


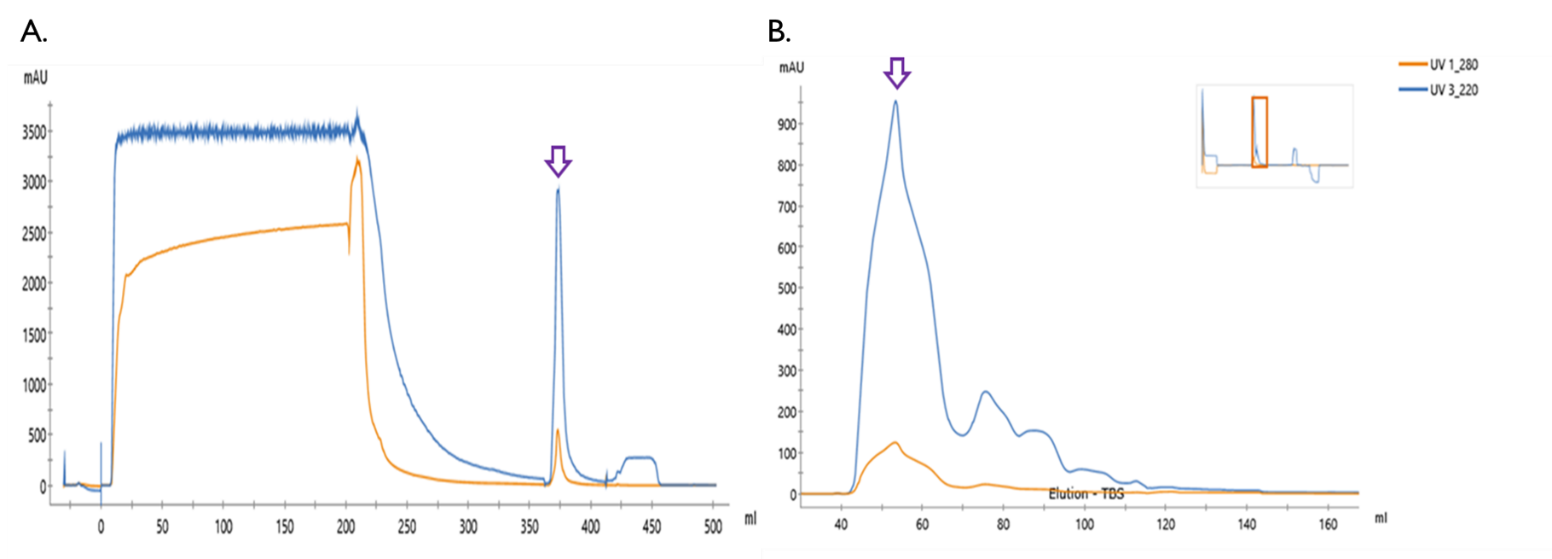


**Supplementary Figure 1: Representative chromatograms from (A) c-tag affinity and (B) size-exclusion chromatography.** Purple arrows indicate the UV peaks for the purified material during elution step.


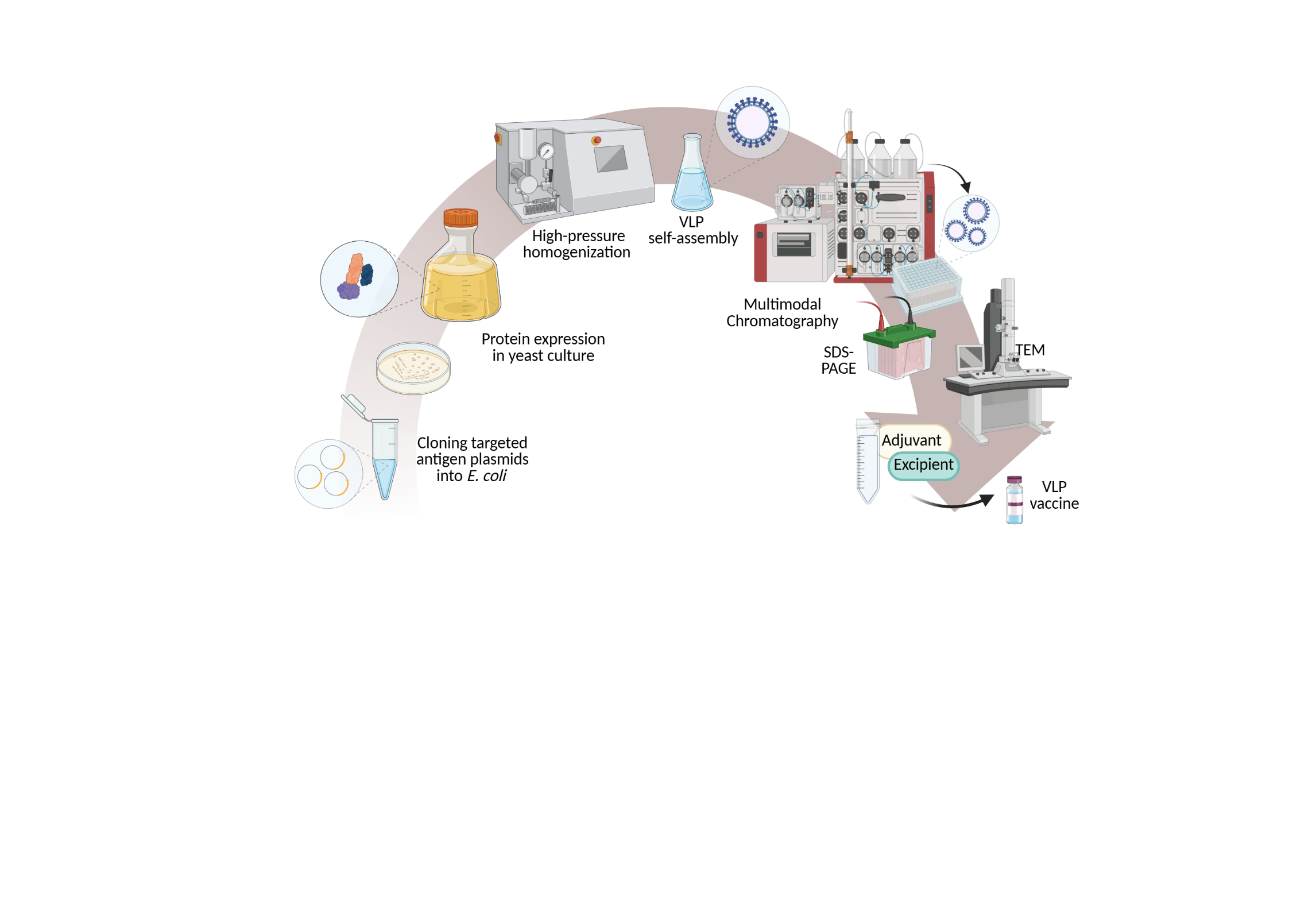


Supplementary Figure 2: Quick summary of VLP production workflow. Recombinant constructs encoding malaria antigens were cloned into *E. coli* for quick expression. Then plasmid of interest was cloned via restriction enzymes and transformed into yeast cells after linearization. After the transformation, the yeast cells were grown, and isolated colonies were induced and screened for the presence of the target sequence. Once a positive clone was identified, it was expanded and induced to produce the protein intracellularly. The cells were then harvested and lysed via high-pressure homogenizer. Size exclusion chromatography was used to acquire the purified product after the lysate was first purified using affinity chromatography with C-tag. Subsequently VLPs were analyzed for structural integrity and antigen display. Downstream characterisation included biochemical and biophysical analyses, as well as immunological assays. Purified VLPs were then formulated with selected adjuvants prior to *in vivo* administration. Figure created via BioRender.com.


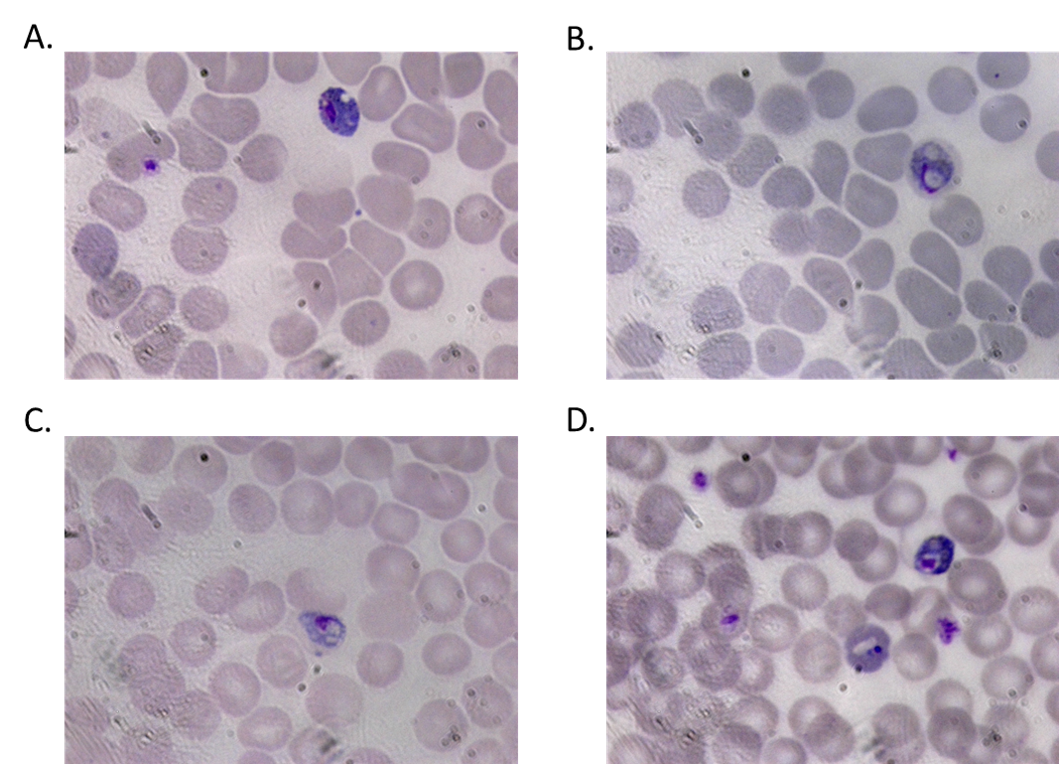


Supplementary Figure 3: Female (A, C) and male (B, D) gametocytes of *P. berghei* transgenic parasite, photos taken from the challenge study monitored by thin blood films, Giemsa staining.


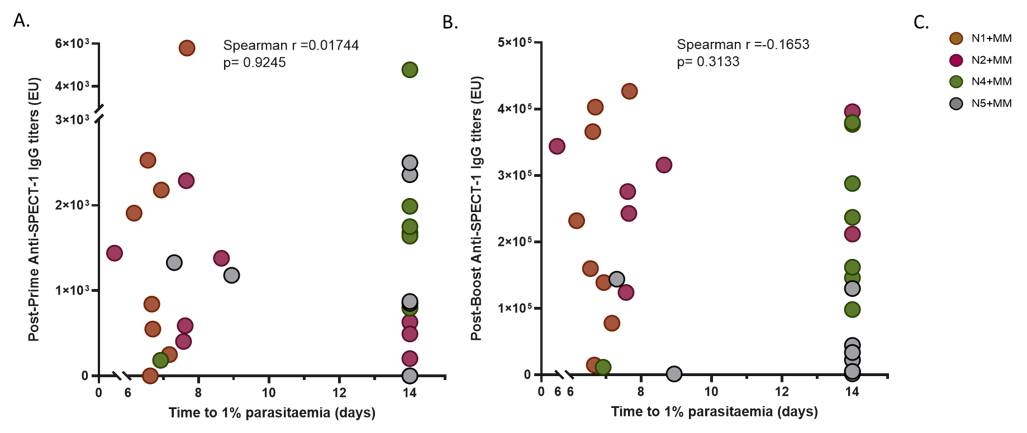


**Supplementary Figure 4: (A) Post-prime and (B) post-boost anti-SPECT-1 IgG titers does not show any correlation with time to 1% parasitemia.** For protected samples, parasite count of 1 was assigned for statistical analysis purposes. All three groups of bivalent and the N1 group samples were combined for the analysis. Animals that remained parasitemia-free throughout the study were assigned the maximum follow-up time (day 14). Correlation analysis was performed using Spearman’s method. A value of p=0.05 or less was considered statistically significant for all analyses and displayed with asterisks (*=p≤ 0.05, **=p≤ 0.01, ***=p≤ 0.001, and ****=p≤ 0.0001).


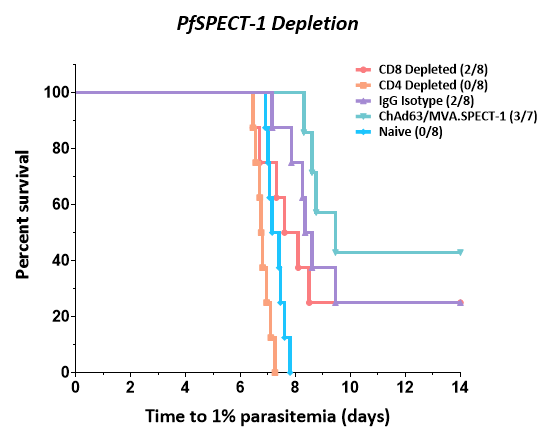


Supplementary Figure 5: *In vivo* depletion of CD4^+^ and CD8^+^ T cells prior to challenge. Mice were immunized with ChAd63/MVA.SPECT-1 and subsequently challenged with transgenic *P. berghei* parasites. To assess the contribution of T cell subsets to protection, animals were treated with depleting antibodies against CD4 or CD8 on days −2 and −1 prior to challenge and on the day of challenge. Control animals received isotype-matched IgG. Depletion efficiency was confirmed by flow cytometry of peripheral blood mononuclear cells. Protection was reduced following CD4+ T cell depletion, whereas CD8+ T cell depletion did not alter the outcome, indicating a role for CD4+ but not CD8+ T cells in this model. Data are presented as survival curves from 8 mice per group.
